# A neurophenomenological model for the role of the hippocampus in temporal consciousness. Evidence from confabulation

**DOI:** 10.3389/fnbeh.2015.00218

**Published:** 2015-08-26

**Authors:** Gianfranco Dalla Barba, Valentina La Corte

**Affiliations:** ^1^INSERMParis, France; ^2^Département de Neurologie, Institut de la Mémoire et de la Maladie d’Alzheimer (IM2A), Hôpital de la Salpêtrière,Paris, France; ^3^Dipartimento di Scienze della Vita, Università degli Studi di TriesteTrieste, Italy; ^4^Inserm U 1127, CNRS UMR 7225, Sorbonne Universités, UPMC Univ Paris 06 UMR S 1127, Institut du Cerveau et de la Moelle épinière, ICM, F-75013Paris, France

**Keywords:** memory, amnesia, confabulation, consciousness

## Abstract

Confabulation, the production of statements or actions that are unintentionally incongruous to the subject’s history, background, present and future situation, is a rather infrequent disorder with different aetiologies and anatomical lesions. Although they may differ in many ways, confabulations show major similarities. Their content, with some minor exceptions, is plausible and therefore indistinguishable from true memories, unless one is familiar with the patient’s history, background, present and future situation. They extend through the whole individuals’ temporality, including their past, present and future. Accordingly, we have proposed that rather than a mere memory disorder; confabulation reflects a distortion of Temporal Consciousness (TC), i.e., a specific form of consciousness that allows individuals to locate objects and events according to their subjective temporality. Another feature that confabulators share is that, regardless of their lesion’s location, they all have a relatively preserved hippocampus (Hip), at least unilaterally. In this article, we review data showing differences and similarities among forms of confabulation. We then describe a model showing that the hippocampus is crucial both for the normal functioning of TC and as the generator of confabulations, and that different types of confabulation can be traced back to a distortion of TC resulting from damage or disconnection of brain areas directly or indirectly connected to the hippocampus. We conclude by comparing our model with other models of memory and confabulation.

## Introduction

Confabulation is a kind of memory distortion, that, at a general level, can be defined as the production of statements or actions that are unintentionally incongruous to the subject’s history, background, present and future situation (Dalla Barba, 1993a; Dalla Barba et al., 1997b). Classically described in Korsakoff’s syndrome, following lesions of the mammillary bodies and the dorsomedial nucleus of the thalamus (TH), and often present after lesions to the orbitofrontal cortex (OFC), confabulation is observed in several conditions affecting the nervous system and follows lesions located in more than 20 anterior and posterior brain areas (Dalla Barba and Boissé, 2010).

Since the early description of this phenomenon, clinicians and scientists have distinguished between different forms of confabulation (Bonhoeffer, 1904; Berlyne, 1972; Dalla Barba, 1993a; Schnider, 2008). One of the most influential distinctions between types of confabulation is the one proposed by Kopelman (1987) between provoked and spontaneous confabulations. Like other distinctions, the one proposed by Kopelman shows advantages and limits. The advantage is that it provides a separation between phenomena that may reflect differing underlying cognitive and neural mechanisms. The limit is that it fails to classify a number of confabulations that are not appropriately captured by either of the distinction’s terms. It has been proposed and used elsewhere a new taxonomy of confabulation, showing that, regardless their modality of appearance, provoked *vs.* spontaneous, confabulations are plausible memories, mainly reflecting the recall of repeated personal events mistakenly considered by the confabulating patient as specific and unique events that occurred in a specific and unique temporo-spatial context. This, by far the more frequent type of confabulation, was named “Habits Confabulation” (Dalla Barba and Boissé, 2010; La Corte et al., 2010) and it was traced back to the disruption of the cognitive mechanism that allows individuals to discriminate between “uniqueness”, i.e., specific unique events, and “multiplicity”, i.e., repeated events, habits and routines (Serra et al., 2014). In these studies, “bizarre”, “implausible”, “fantastic” confabulations, either spontaneous or provoked represented less than 5% of the total number of confabulations.

Although they may differ in many ways, confabulations show major similarities:

1. Their content, with some minor exceptions, is plausible and therefore indistinguishable from true memories, unless one knows the patient’s past, present and probable future situation (Dalla Barba, 1993a).
2. They extend through the whole individuals’ temporality, including their past, episodic memory, their orientation in their present world and their ability to predict their personal future (Dalla Barba et al., 1990, 1997b, 1999; Dalla Barba, 1993a; Nedjam et al., 2000; Schnider, 2008; La Corte et al., 2010). Accordingly, Dalla Barba (2002) proposed that rather than a mere memory disorder, confabulation reflects a distortion of Temporal Consciousness (TC), i.e., a specific form of consciousness that allows individuals to have phenomenological experience of remembering their personal past, of being oriented in their present world and of predicting their personal future.
3. It is uncontroversial that patients with documented, complete, bilateral hippocampal damage are amnesics, but don’t confabulate, whereas, regardless of their lesion’s location, confabulators all have a relatively preserved hippocampus, at least unilaterally (Gilboa and Moscovitch, 2002; Dalla Barba and Boissé, 2010).

Accordingly, Dalla Barba and La Corte (2013) proposed a model in which the hippocampus is the neural correlate of TC, which is lost in hippocampal amnesia and malfunctioning in confabulation.

According to the model they proposed, lesions occurring to brain areas and pathways upstream or downstream an intact or partially preserved hippocampus produce different types and possibly different modality of appearance of confabulation. In that model the hippocampus plays a passive role receiving directly from upstream pathways, or indirectly, through the cingulum and the retrosplenial cortex already distorted information. The aim of the present work is to further develop the previous model. In this new perspective, a partially damaged hippocampus may still allow TC to exist, but may loose its peculiarity of segregating and organizing information in the temporo-parietal cortex (TPC).

In this article, we review data showing differences and similarities among forms of confabulation. We then develop the model sketched in our previous work (Dalla Barba and La Corte, 2013) showing that the hippocampus is crucial both for the normal functioning of TC and as the generator of confabulations, and that different types of confabulation can be traced back to a distortion of TC resulting from damage or disconnection of brain areas directly or indirectly connected to the hippocampus.

The model described in this work is a “neuro-phenomenological” one, in the sense that it combines the phenomenological description of confabulation and neurological or neurocognitive experimental accounts of the issues treated in this work.

## Varieties of Confabulation: Differences and Similarities

### Differences in Etiology and Anatomy

Confabulation is a rather infrequent disorder with different aetiologies and anatomical lesions. It is a pathognomonic sign of Korsakoff’s syndrome (Korsakoff, 1889; Bonhoeffer, 1904; Wyke and Warrington, 1960; Talland, 1961; Mercer et al., 1977; Cermak et al., 1980; Dalla Barba et al., 1990; Benson et al., 1996; Schnider et al., 1996a), but is observed also in other pathological conditions, namely in patients suffering from ruptured aneurism of the anterior communicating artery, subarachnoid haemorrhage or encephalitis (Luria, 1976; Stuss et al., 1978; Kapur and Coughlan, 1980; Alexander and Freedman, 1984; Moscovitch, 1989, 1995; Delbecq-Derouesné et al., 1990; DeLuca and Cicerone, 1991; Irle et al., 1992; Kopelman et al., 1995; Papagno and Muggia, 1996; Schnider et al., 1996b; Dalla Barba et al., 1997b; Diamond et al., 1997), head injury (Weinstein and Lyerly, 1968; Baddeley and Wilson, 1986; Dalla Barba, 1993b), Binswanger’s Encephalopathy (Dalla Barba, 1993a), Alzheimer’s disease (AD) and frontotemporal dementia (Kern et al., 1992; Dalla Barba et al., 1999; Nedjam et al., 2000, 2004; Attali et al., 2009) and aphasia (Sandson et al., 1986). On occasion, or in particular experimental conditions, confabulation may also be observed in normal subjects (Kopelman, 1987; Burgess and Shallice, 1996b; Dalla Barba et al., 2002).

At a general level, distinctions between types of confabulation are considered to reflect different underlying brain lesions. Anterior brain lesions, in particular in the OFC basal forebrain and related structures, have been consistently associated with spontaneous confabulations (Kopelman, 1987; Moscovitch, 1995; Schnider et al., 1996a; Schnider and Ptak, 1999; Gilboa et al., 2006b). Spontaneous confabulations, however, can also occur with lesions not involving the OFC and related structures (Dalla Barba, 1993a; Dalla Barba and Boissé, 2010; La Corte et al., 2011). Other types of confabulation lack a specific anatomical basis, but are usually related to posterior cortical and subcortical lesions sparing the OFC and related structures. Overall, lesions in more than 20 brain regions have been reported in confabulation (Gabrieli et al., 1988; Gilboa and Moscovitch, 2002; Dalla Barba and Boissé, 2010). Therefore, unlike many other neuropsychogical disorders, e.g., aphasic syndromes, confabulation is not associated with a specific lesion site.

### Similarities in Content

Regardless of their etiology and anatomy, most confabulations are indistinguishable from true memories, in the sense that their content is plausible, mostly consisting of habits, repeated events or over-learned information mistakenly considered as specific unique episodes, so that an observer blind to the patient’s past, present and future situation wouldn’t be able to tell whether the patient is confabulating or not (Dalla Barba, 1993a; Dalla Barba et al., 1997b, 1999; Burgess and McNeil, 1999; La Corte et al., 2010). The case of patient MG (Dalla Barba et al., 1997a) well illustrates how confabulations can go undetected when the real present and past situation of a patient is unknown. While he was waiting to undergo a CT scan, MG told the radiologist that he had accompanied a friend to be admitted to the neurology department that day. The neurologist who was taking care of MG’s (inexistent) friend realized that MG also had neurological problems and so decided to refer him to the radiology department for a CT scan. On that occasion the radiologist did not even suspect that MG was confabulating. Another example is the following:

– How did your disease begin? Asks the doctor to patient CA.
– It started with a strong sore throat … I couldn’t swallow anything … so I couldn’t go to school … my mother called the doctor, answers the patient.
– How did your disease begin? Asks the same doctor to another patient, CD.
– It started with a strong headache … one morning I woke up with a strong headache and then I started throwing up and I remember I couldn’t keep my eyes open, answers the patient.

One of these two patients is confabulating, whereas the other one is reporting the memory of an event she has really experienced. As you can see, there is nothing in the patients’ reports that can help you to tell which patient is confabulating. However, if we tell you that CA is a 67-year-old woman with Korsakoff’s syndrome (Dalla Barba et al., 1990) and that CD is a 33-year-old woman reporting the onset of her Herpes meningitis, things become much more clear and these additional pieces of information allow you to identify CA as the confabulating patient. This is not because you know that patients with Korsakoff’s syndrome confabulate, but rather because you know that it is quite unlikely that somebody who is 67-year-old goes to school and has a mother who calls the doctor for her sore throat. In addition you know that headache, vomiting and photophobia are common in the onset of Herpes meningitis, which suggests you that CD is not confabulating. Nevertheless, CA’s and CD’s reports have something in common. They are plausible (Dalla Barba, 1993a) in the sense that an observer blind to the patient’s personal past wouldn’t be able to tell whether the patient is confabulating or not.

### Temporal Similarities

However, the confabulators’ tendency to mistake habits and repeated events as unique episodes encompasses not only their personal past, but involves their personal present and future as well. In fact, they often confabulate about their present situation (Dalla Barba et al., 1990, 1998; Dalla Barba, 1993a; Burgess and McNeil, 1999; La Corte et al., 2010, 2011) saying, for example, that they are at school rather than at the hospital (Dalla Barba et al., 1990), and make confabulating errors concerning their personal future, saying, for example, that the following day they will be going at work, although they are not working anymore (Burgess and McNeil, 1999; Schnider, 2008; La Corte et al., 2011). Sometimes they also act upon their confabulated present and future. Patient MB (Dalla Barba, 1993a), for example, on one occasion said that he was looking forward to the end of the testing session because he had to go to the general store to buy some new clothes, since he hadn’t been able to the day before. On this occasion the patient actually attempted to leave his hospital room, claiming that there was a taxi waiting for him downstairs. The patient’s tendency to confabulate in the three dimensions of personal temporality—past, present and future—has been consistently reported using the Confabulation Battery (Dalla Barba, 1993a; Dalla Barba and Decaix, 2009) in 20 patients with confabulatory syndromes of various aetiologies and with different brain lesions (Dalla Barba et al., 1997a, b; Dalla Barba and Boissé, 2010; La Corte et al., 2010, 2011).

### Anatomical Similarities

Regardless the lesions’ heterogeneity, confabulators have at least partial preservation of the hippocampus (used here to refer to the hippocampus proper together with the dentate gyrus and the subicular cortex). In a review of 79 cases of confabulation, it was found that none of these patients had hippocampal lesions (Gilboa and Moscovitch, 2002). Other 28 confabulators, not considered in the above review, also had normal hippocampi (Dalla Barba et al., 1990, 1997b; Dalla Barba, 1993a; Fotopoulou et al., 2004, 2007; Ciaramelli et al., 2006; Ciaramelli and Ghetti, 2007). Damage to the hippocampus has long been known to produce amnesia (Scoville and Milner, 1957), i.e., a retrograde and anterograde episodic memory deficit “out of all proportion to other memory and cognitive functions in an otherwise alert and responsive patient” (Victor et al., 1971). Episodic memory dysfunction varies according to the degree of hippocampal damage. In early AD, mild to moderate hippocampal atrophy induces mild to moderate episodic memory deficit. Episodic memory is completely abolished following complete, bilateral hippocampal damage. Amnesic patients show normal or close to normal performance on a number of implicit learning and memory tasks, have preserved linguistic skills and have relatively preserved general knowledge or semantic memory. In contrast, they are completely unable to learn and retain any new information, show extensive retrograde amnesia, and have no phenomenological experience of remembering their personal past and of predicting their personal future. In these patients, who are sometime described as stucked in an instantaneous present, the three dimensions of personal temporality, past, present and future, are lost. They have no difficulties with physical or chronological time (Husserl, 1893). They have preserved semantic knowledge of units of time and their relationships (Tulving, 1985). They have relatively preserved knowledge of past public and historical events and they can predict episodes and events in the public domain (Klein et al., 2002). But in contrast with this preserved knowledge of physical and impersonal time, their awareness of subjective time is severely impaired. Accordingly, classic hippocampal amnesia cannot be considered a pure episodic memory deficit, but rather a pathological condition affecting individuals’ episodic subjective temporality.

Within the framework of the Memory, Consciousness and Temporality Theory (MCTT; Dalla Barba, 2002), it has been proposed that confabulation reflects a distortion of TC, whereas classic amnesia due to hippocampal damage reflects a loss of TC.

## The Memory, Consciousness and Temporality Theory (MCTT)

In line with the continental phenomenological tradition (Brentano, 1874; Sartre, 1943; Merleau-Ponty, 1945; Husserl, 1950), the MCTT considers that consciousness is not an aspecific entity, but is intentionally projected toward its object, being always consciousness of *something*. Here and hereafter the object of consciousness is not meant to be necessarily a physical object, but it is what consciousness is addressing, a physical object, e.g., a pen, or an abstract object, e.g., an event. Consciousness addresses its object in different ways, implying that different types, or modes of consciousness exist. For example, this pen in front of me on the desk, I can perceive it, if I close my eyes, I can imagine it, I can hate it or like it, I can know it, e.g., know that is it is a pen and not a sailing boat, I can remember it, e.g., remember where and when I bought it. All these different relationships between consciousness and its object are *original*, because each one differs from each other and *irreducible*, because they are not the final result of a causal or ontological cascade. The aim of this work is not to detail a taxonomy of different types of consciousness, but to use the distinction made by Dalla Barba (2002) between TC and Knowing Consciousness (KC).

KC is defined as a specific form of consciousness allowing individuals to be aware of past, present and future *impersonal* knowledge and information. KC concerns, for example, knowing that G. W. Bush was the past President of the United States, that Obama is currently in charge of that position and that in the next Presidential elections he will be not allowed to run for a third term. KC is usually relatively preserved in both confabulating and non confabulating amnesics (Dalla Barba et al., 1997b; Klein et al., 2002; Dalla Barba and Boissé, 2010; La Corte et al., 2010, 2011). Patients who have no phenomenological experience of remembering their personal past and of predicting their personal future not only are able to retrieve impersonal past information, i.e., semantic memories, but are also able to predict the impersonal future. For example, they have no difficulties in answering questions like “What is likely to be an important breakthrough in the medical domain in the next 10 years?” (Klein et al., 2002; La Corte et al., 2010, 2011). They also have preserved “personal semantics”, i.e., they have access to personal past and present factual information. They can correctly give, for example their date of birth and they can tell that they went to school and then graduated. They can also use this information to make inferences about their future. Yet none of these cases do they have the phenomenological experience of remembering specific episodes from their personal past and of predicting specific episodes in their personal future.

TC is a specific form of consciousness that allows individuals to have phenomenological experience of remembering their personal past, of being oriented in their present world and of predicting their personal future (Dalla Barba and La Corte, 2013). It is this type of consciousness that defines individuals as temporal entities with a personal past, present and future. Personal temporality, as expressed by TC, is different from impersonal temporality, as expressed by KC. Patients without TC, due to bilateral hippocampal damage (see below) still have impersonal temporality, i.e., they can access and use impersonal past, present and future information, but they have lost the personal dimension of time. They can learn and *know* things about their past, as they can* know* things about their future. They can even learn their entire biography (see below the description of patient RM), but they have no phenomenological experience of remembering their past and of projecting themselves in specific future situations.

In normal conditions, TC addresses the object’s *Uniqueness* (U), whereas KC addresses the object’ *Multiplicity* (M). Let’s consider the following example. This pen on the desk reveals both an U and a M, according to how my consciousness address it. It represents a U if I consider it *that-specific-pen-I-bought-last-week-and-that-I-will-be-using-this-afternoon-to-sign-a-cheque*. It represents a M if I consider it *an-indeterminate-pen-belonging-to-the-category-of-pens*. In short, U means *this specific pen* and not another one, whereas M means *a pen*, an object belonging to the multiplicity of objects of the same category. Accordingly to how consciousness addresses its object, the pen in this example, the object reveals a U, or a M. Anticipating what will be discussed later on, in normal conditions, TC’s object represents U, whereas KC’s object represents M.

In the next section, we will see how what we have discussed so far is relevant to the interpretation of confabulation.

## Temporal Consciousness, Confabulation and Amnesia

In confabulators, TC is present, but it is malfunctioning, because these patients confabulate when questioned about their past, present and future. Conversely, non-confabulating amnesics, who have lost TC, have no phenomenological experience of remembering their personal past and of predicting their personal future. An increasing number of studies have addressed the question of the neurocognitive relationship between episodic memory and the individuals’ ability to predict their personal future. What is referred to, as memory of the future (Ingvar, 1985), planning (Dalla Barba et al., 1997b) or imagining the future (Klein et al., 2002; Schacter et al., 2007), mental time travel (Suddendorf and Corballis, 2007) and chronesthesia (Tulving, 2002) are aspects of TC as described in the MCTT (Dalla Barba, 2002). Schacter et al. (2007) propose that remembering the past is necessary to imagine the future. However, although remembering the past and imagine the future depend much on the same neural machinery, namely the medial temporal lobe, there is no ontological priority of remembering vs. predicting the future. In other words remembering is not a prerequisite to predict the future. It is not because I remember that I had a cup of thee this morning that I am able to predict having sushi for dinner tonight.

As reported elsewhere by Dalla Barba and co-workers (Dalla Barba and Boissé, 2010; Dalla Barba and La Corte, 2013), aspects of the MCTT relevant to the interpretation of confabulation and amnesia are summarized below.

1. Events produce atemporal and aspecific patterns of modifications in the brain. These modifications, are atemporal in the sense that they do not contain any information concerning time. They do not represent the past, the present or the future, nor are they organized according to the order of succession, i.e., there is nothing in Y, for example, that tells that Y come before Z and after X. They are aspecific in the sense that they do not contain any information specifying that they are representing episodes, meanings, rules, procedures, or algorithms.
2. The patterns of modifications in the brain can be more or less stable and more or less vulnerable depending on a number of variables. These variables include, among others, attention at encoding, emotional value of the event, depth of encoding, rehearsal and repeated experience of the same or of a similar event.
3. TC is a specific form of consciousness that allows individuals to have phenomenological experience of remembering their personal past, of being oriented in their present world and of predicting their personal future. It is specific because it cannot be confounded with other forms of consciousness, such as, for example, perception and imagination. If, for instance, the average person perceives a tiger in front of them, they will be scared, but if they remember or imagine such an event, they may not be scared at all.
4. TC is experimentally measurable and dissociable from impersonal temporality. Using the Confabulation Battery, which includes 11 dependent variables (Dalla Barba, 1993a; Dalla Barba and Decaix, 2009), it has been demonstrated that confabulators and amnesics either confabulate or have no phenomenological experience of remembering their personal past and of predicting their personal future, whereas they are able to answer questions about impersonal past, present, and future (e.g., what happened to Princess Diana, who the President of the United States is and what is likely to be one of the most important breakthrough in the medical domain in the next 10 years; La Corte et al., 2011; Klein et al., 2002).
5. TC is lost in amnesia following complete bilateral hippocampal damage and malfunctioning in confabulation, because it receives distorted information from more than 20 damaged, predominantly orbitofrontal, brain areas.
6. The object of consciousness represents a determination and an indeterminateness, what we have called U and M. U refers to unique events, whereas M refers to repeated events. TC addresses the object’s U, whereas KC addresses its M.

In normal conditions, TC interacts with less stable patterns of modification of the brain in order to seize the object’s U, past, present or future, whereas KC, interacts with more stable patterns of modification of the brain in order to seize the object’s M. The interaction between TC and less stable patterns of modification of the brain allows individuals to identify the “pen” as a U, i.e., as an object belonging to a personal temporality—I have used this pen yesterday to sign a cheque, it is now in front of me, just some inches beside the computer’s keyboard, and I can predict using it tomorrow to sign another cheque for the plumber. In contrast, the interaction of KC with more stable patterns of modification of the brain allows people to identify the “pen” as a M, i.e., as a specific object, which is different from other objects—this pen in front of me is different from the computer’s keyboard, although they share similar functions.

In amnesia TC is lost. Non-confabulating amnesic patients don’t have any phenomenological experience of remembering or of predicting specific unique events in their personal past or personal future. They can recognize elements of their life as familiar, but this, in the framework of the present theory, does not reflect uniqueness. They can say: “this is my dog, my mother, my car, my house”, but they don’t have any phenomenological experience of remembering or of predicting any specific unique episode concerning their dog, mother, car or house. In other words, since they have lost TC, they have lost the possibility of segregating specific episodic information within a network of information, which is the necessary condition to access objects’ uniqueness (see below).

In confabulation, TC is still present, but it is not interacting with less stable patterns of modification of the brain, because these modifications are abolished or inaccessible in the mode of TC. In this condition, TC interacts with more stable patterns of modifications of the brain, and the result is that repeated events, habits and over-learned informations, in short the object’s multiplicity, are seized as unique events, past, present or future. It is clinically well known, for instance, that hospitalized confabulators, when directly questioned on what they have done the previous day, usually report routine activities from their life before the accident. For example, they may say that the previous day they went to work or that they had dinner at home “as usual”. In this case, irretrievable episodic memories, i.e., events that occurred in a unique and specific temporo-spatial context, are replaced by routines, i.e., multiple, repeated events that didn’t occur in a unique and specific temporo-spatial context. Therefore we can say that M, i.e., routines and repeated events, is mistaken for U, i.e., a specific unique event that occurred in a specific, unique temporo-spatial context (such as the previous day). This clinically well known observation has been experimentally demonstrated for the first time in a recent work from the Dalla Barba’s group (Serra et al., 2014). In order to measure the ability to discriminate unique from repeated events the authors used four runs of a recognition memory task, in which some items were seen only once at study, whereas others were seen four times. Confabulators, but not non-confabulating amnesics, considered repeated items as unique, thus mistaking M for U. The authors suggested that a crucial mechanism involved in the production of confabulations is thus the confusion between unique and repeated events.

It might be argued that this account may explain Habits Confabulations, but not other types of plausible confabulations, which do not necessarily arise from the patient’s own life. The example of patient MG and the radiologist described earlier in this paper reports a plausible confabulation, but there is no evidence that the patient ever went visiting a friend admitted to a neurology department. However, Habits Confabulations, the most common form, and other types of plausible confabulation may rely on very similar mechanisms involving the hippocampus ability to segregate and organize information in the temporo-parietal associative cortex. In the Dalla Barba and La Corte (2013) model the hippocampus played a sort of “passive” role. It passively received distorted information directly from the TPC or indirectly, through the cingulum, and “temporalized” them in a personal temporal framework. The result of this condition is that the hippocampus produces confabulation because it receives distorted information from upstream or downstream from other brain areas it is connected with. In the Dalla Barba and La Corte (2013) model, the hippocampus is a brain structure that receives distorted information and locates them in a personal temporal framework. However, it is known and accepted (e.g., Hardt et al., 2013) that the hippocampus has also an active role. It acts as a sort of “pointer”, making a fine-grained search in the neocortex segregating specific episodic information within a network of information, which is not (necessarily) pertinent to the goal, i.e., the retrieval of specific episodic memories. If the hippocampus is partially damaged, it may select plausible information based on the patient’s habits. However, if plausible habits are unavailable, or do not fit the current demands, it may make an “abductive inference” (Coltheart et al., 2010), providing the best plausible explanation of the patient’s current situation.

In the La Corte et al. (2010) study “bizarre”, “implausible”, “fantastic”, “semantically anomalous” confabulations, either spontaneous or provoked represented less than 5% of the total number of confabulations. The model proposed here accounts for this type of confabulation. Lesions upstream the hippocampus may produce deep semantic deficits, which may produce “semantically anomalous” confabulation (Dalla Barba, 1993b), i.e., confabulations with semantically incoherent content. Lesions downstream the hippocampus, in particular in the OFC may produce “fantastic” or “bizarre” confabulations. It is well known that patients with orbitofrontal lesions often show inadequate and bizarre behavior. This type of behavior may extend to the memory domain and to the domain of TC in general.

In contrast to confabulation, in amnesia, due to complete hippocampal damage, TC is lost. Patients with classical amnesia are unable to temporalize objects. They can’t remember their past, they are disoriented in the present world and they are unable to prospect their future. Since in these patients TC is lost, no interaction is possible between TC and more or less stable patterns of modification of the brain. In contrast, in these patients, KC is relatively preserved and interacts normally with more stable patterns of modification of the brain. Therefore they can access and use impersonal information concerning the past, the present and the future. They can say, for example, that Kennedy was killed, that France is a republic, whereas UK is a kingdom, and that the candidate for the Democrats in the next US Presidential elections will not be Barack Obama.

## Neural Correlates of Temporal Consciousness

So far we have seen that what distinguishes confabulators from non-confabulating amnesics is a distorted TC, in the first, and a loss of TC, in the latter. We have also seen that available data indicate that the integrity, at least partial or unilateral, of the hippocampus seems to be a necessary condition in order for individuals to confabulate, whereas its complete damage not only is not associated with confabulation, but results in a loss of TC, and consequently in deep amnesia.

It is known that some patients with bilateral lesions in the hippocampus confabulate. Patients with limbic encephalitis (Kikuchi et al., 1999; Nahum et al., 2010; Kartsounis and de Silva, 2011) and Pick’s disease with hippocampal involvement (Kremen et al., 2010), for example, confabulate. But limbic encephalitis and Pick’s disease don’t lead to complete, bilateral hippocampal destruction. In the Nahum et al. (2010) study, inflammation was pronounced in the left hippocampus, but was only mild in the right one. In the Kremen et al. (2010) study, it is clearly stated that the hippocampus was relatively spared bilaterally. One case is reported to have complete limbic lobe destruction and confabulation (Gascon and Gilles, 1973). However, in this patient complete, bilateral hippocampal damage is not documented. Overall, there is overwhelming evidence supporting the conclusion that at least partially preserved hippocampus is a necessary condition for confabulation.

At variance with patients with hippocampal amnesia, patients with diencephalic amnesia have distorted TC, as defined in this and previous work from Dalla Barba and co-workers. Confabulation, which is the hallmark of a distorted TC, is a pathognomonic sign of Korsakoff’s syndrome (Korsakoff, 1889; Bonhoeffer, 1904; Wyke and Warrington, 1960; Talland, 1961; Mercer et al., 1977; Cermak et al., 1980; Dalla Barba et al., 1990; Benson et al., 1996; Schnider et al., 1996a; Borsutzky et al., 2008), which is a diencephalic amnesia. Patients with non-Korsakoff thalamic lesions (e.g., Gentilini et al., 1987; Hodges and McCarthy, 1993; Markowitsch et al., 1993; Markowitsch, 2008) and patients with orbitofrontal lesions (e.g., Kopelman, 1987; Knight et al., 1995; Moscovitch, 1995; Schnider et al., 1996a; Dalla Barba et al., 1997b; Schnider and Ptak, 1999; Gilboa et al., 2006b) show deep anterograde, more variably, retrograde amnesia and, invariably, various types of memory distortions, i.e., distorted TC, including confabulations.

Taken together, these observations show that hippocampal amnesia, complete bilateral destruction of the hippocampus, produces negative signs and symptoms, i.e., the failure to retrieve the desired information in TC, whereas non-hippocampal amnesia, diencephalic and frontal, produce positive signs such as memory distortions. This strongly suggests that the hippocampus is the neural correlate of TC (Dalla Barba and La Corte, 2013) and is supported by an increasing number of recent neuropsychological (Klein et al., 2002; Hassabis et al., 2007; Rosenbaum et al., 2007; Kwan et al., 2010) and neuroimaging (Martin, 2001; Schacter and Addis, 2007; Botzung et al., 2008; Addis et al., 2011) studies confirming that the hippocampus is a core structure within a network involved in individuals’ temporal existence, i.e., their having phenomenological experience of a personal past, present and future.

Hippocampal anatomy, physiology and connectivity are all suggestive of a crucial function of this neural structure in associating experienced events in order to remember specific episodes from one’s own past and to adapt to ongoing and future reality (Henke, 2010). The hippocampus is reciprocally connected, either directly or indirectly, with all neocortical association areas. It receives upstream, through the parahippocampal, perirhinal, and entorhinal cortices, projections from unimodal and polymodal neocortical association areas and projects downstream, through the fornix, to the hypothalamus, the anterior thalamus, the anterior cingulate gyrus and the OFC. Lesions to the fornix result in amnesia without confabulation, whereas confabulation has been described for lesions involving all the above neural structures, but sparing the hippocampus.

If the hippocampus is the neural correlate of TC, then its function is to *temporalize* information. This doesn’t mean that the hippocampus has a subjective intentional life, like monitoring theories assume for the, anthropomorphised, frontal lobe (see Dalla Barba, 2002 for a discussion of the *homunculus* fallacy and the anthropomorphisation of the brain), but that information, normal or distorted, assumes a personal, temporal dimension when processed by the hippocampus. In normal conditions, the hippocampus accomplishes its function very well, being able to capture the events’ uniqueness—that specific walk I had yesterday afternoon along the Bastille Canal and not the walk I take each day there, or the conference I will give tomorrow at 5 pm and not a general talk I will be giving in the future. It is now well known that the hippocampus is crucial for rapid, single-trial learning of flexibly integrated what-where-when information (Henke, 2010). Consistently, it has been shown that long-term potentiation following a single train of high-frequency tetanic stimulation can be induced in the hippocampus (Trepel and Racine, 1998). Thus, the normal function of the hippocampus is to temporalize unique phenomenological experiences. This function is the result of the interaction between the hippocampus and less stable patterns of modification in neocortical unimodal and polymodal association areas.

As we have seen, in keeping with some aspects of the MCTT, these patterns of modification of the neocortical association areas are atemporal and aspecific. They are atemporal because they do not represent the past, the present or the future. They are aspecific because they do not contain any information specifying that they are representing episodes, meanings, rules, procedures, or algorithms. These patterns of modifications of the neocortical areas are made temporal and specific by the hippocampus, which processes them as temporal and specific. The patterns of modification that events produce in the neocortical areas can be expressed in behavior atemporally and aspecifically, like, for example, in priming effects. In priming effect, single, unique past events influence current performance and behavior without being temporalized, i.e., they are not expressed as elements of a personal past, present or future. This is known since the pioneering clinical reports by Korsakoff, Claparède and others.

So, at present, the hippocampus is the best candidate as the neural correlate of TC, although it is involved in other functions, like reaction to novelty, single trial learning and “unconscious” episodic memory (Henke, 2010).

## Hippocampus Confabulation and Amnesia

In its classical form, confabulation is observed for lesions in the mammillary bodies and the dorsomedial thalamic nucleus. It is well known that confabulation is frequently observed following lesions in the OFC and basal forebrain. These uncontroversial observations lead researchers to consider lesions to these neural structures as crucial for confabulation to occur. However, as mentioned above, confabulations are observed for lesions in more than 20 anterior and posterior cortical and subcortical areas, which are all directly or indirectly connected to the medial temporal lobe and to the hippocampus. The OFC is one of these structures to which the hippocampus projects through the fornix, mammillary bodies and TH. Lesions to the mammillary bodies and the TH, but also to the basal forebrain produce confabulations (Schnider, 2008). In short, with the exception of lesions involving the fornix, damage at any point of the pathways running downstream the hippocampus produce confabulation, provided that the hippocampus is, at least partially, preserved. If the hippocampus is severely damaged bilaterally the result is deep amnesia without confabulation (Dalla Barba and La Corte, 2013).

**Figure 1:**
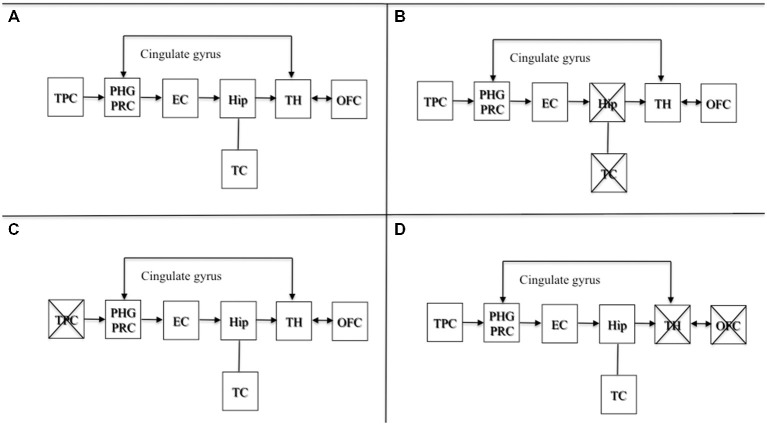
**Adapted with permission from Dalla Barba and La Corte (2013).** A schematic cognitive and neuroanatomical model of normal and pathological functioning of memory and Temporal Consciousness (TC). **(A)** Normal functioning of memory and TC: the hippocampus, which is the neural correlate of TC, temporalizes information received directly from the TPC, or indirectly from the OFC and the TH, through the cingulate gyrus, allowing individuals to remember their personal past, to be oriented in their present and to predict their personal future. **(B)** Amnesia: complete, bilateral lesions to the hippocampus abolish TC, preventing individuals from accessing their personal temporality (i.e., their past, present, and future). **(C)** Implausible or semantically anomalous confabulation: lesions to the TPC provide the hippocampus with distorted semantic information, inducing the hippocampus and TC to make semantically anomalous confabulatory errors concerning the individual’s personal temporality. **(D)** Plausible, or semantically appropriate confabulation: lesions to the TH and the OFC provide the hippocampus with plausible but erroneous information, inducing the hippocampus and TC to make plausible confabulatory errors concerning the individuals’ personal temporality. Abbreviations: TPC, temporo-parietal cortex; PHG, parahippocampal gyrus; PRC, perirhinal cortex; EC, entorhinal cortex; Hip, hippocampus; TH, thalamus; OFC, orbitofrontal cortex; TC: temporal consciousness.

Brain damage involving areas projecting from upstream to a preserved hippocampus are also known to produce confabulation (Dalla Barba, 1993a, b; De Anna et al., 2008; Attali et al., 2009). As stated elsewhere (Dalla Barba and La Corte, 2013), lesions to temporoparietal association areas, or to their projections to the hippocampus, may produce confabulated memories and plans, which may differ in content from confabulations observed from lesions involving the OFC, or structures and pathways downstream of the hippocampus. Lesions downstream of the hippocampus produce semantically appropriate confabulations, either provoked or spontaneous. Lesions upstream of the hippocampal circuit produce more implausible and semantically anomalous confabulations. Therefore, the hippocampus is likely to be the core temporal device that temporalizes personal phenomenological experiences, provided either directly by temporoparietal association areas, or, indirectly, through the Papez’s circuit, by diencephalic, basal forebrain and orbitofrontal structures. Lesions upstream or downstream sparing the hippocampus may all produce confabulation. This model is presented in Figure 1.

Figure 1A depicts the normal functioning of the circuit. Figure 1B shows a complete bilateral damage to the medial temporal lobe and the hippocampus with the consequent loss of TC resulting in deep amnesia. Figure 1C describes lesions to temporo parietal areas or their disconnection to the hippocampus, resulting in semantically anomalous confabulations. Figure 1D shows lesions downstream of the hippocampal circuit producing the most common form of confabulation, which are mainly plausible, semantically coherent and indistinguishable from true memories, unless one is aware of the patient’s past, present and future situation.

The anatomical basis of confabulation has been a puzzling issue. Confabulation was originally described in alcoholic patients (Korsakoff, 1889), later shown to have diencephalic lesions (Victor et al., 1971), but were then observed in patients with chronic infections, traumatic brain lesions, subarachnoid hemorrhage, brain tumors and other pathologies (for a review, see Schnider, 2008). Overall, more than 20 anterior and posterior brain lesion loci have been associated with confabulation (Gilboa et al., 2006a, b; Dalla Barba and Boissé, 2010). Confabulations are also a common finding in diffuse brain pathologies like AD and frontotemporal dementia. Therefore, it seems uncontroversial that confabulation lacks a specific neurobiological correlate, either in terms of pathology, or in terms of lesion’s location. Here, we propose that the neural correlate of confabulation is the, at least partial, integrity of the hippocampus in association with lesions in brain areas that project, directly or indirectly to the hippocampus. Lesions upstream or downstream of the hippocampus may produce different types of confabulation through the disruption of different cognitive processes, but at least a partial integrity of the hippocampus is the necessary condition for confabulation to occur.

As far as the involvement in confabulation of specific regions within the hippocampus is concerned, at present no reliable data are available and consequently, no conclusion is possible. However, it is reasonable to think that CA3 and the posterior hippocampus may be crucial for the normal functioning of TC. Neuroimaging data in normal subjects as well as animal studies show that various long-axis specialisations arise out of differences between the anterior and posterior hippocampus (Poppenk et al., 2013). The anterior hippocampus is involved in coarse, global representations, whereas the posterior hippocampus is involved in fine-grained local representations. CA3 is known to be involved in pattern separation and pattern completion (Rolls, 2013; Deuker et al., 2014). Furthermore, CA3, compared to entorhinal cortex (EC), subiculum, CA1-CA2, is relatively preserved in early AD (Mueller et al., 2007, 2010), a condition in which confabulations are present (Dalla Barba et al., 1999; De Anna et al., 2008; Attali et al., 2009). Fine-grained local representations, pattern separation and pattern completion are processes possibly involved in TC’s recognition of uniqueness (see above). If these processes are disrupted, then multiplicity may be mistaken for uniqueness, because interfering, distorted information from other damaged brain areas or other hippocampal subfields prevents the normal functioning of the posterior hippocampus and CA3, and consequently, of TC. Further research evaluating the role of specific hippocampal subfields in confabulation will provide possible support to what, at present, is mere speculation.

## Comparison with other Models of Memory and Confabulation and Guidelines to the Falsification of the Model

TC, as used here and in other works from the Dalla Barba’s group, is distinct from other types of consciousness, namely KC, Imaginative Consciousness and Perceiving Consciousness (Dalla Barba, 2002). TC is the synthesis of a set of theoretical assumptions, have some specific characteristics and is one of the core concepts of the MCTT (Dalla Barba, 2002). To summarize, TC is:

– the prediction of a concrete personal phenomenon, i.e., individuals’ ability to consciously remember their past, to be consciously oriented in their present world and to consciously predict their personal future;
– experimentally measurable: questions like “Do you remember what you had for dinner last night, the last time you went to the restaurant, the last time you went for a swim?”, or “Can you predict when you will be going to the restaurant, for a swim next time?” are measures of TC. The possibility to answer these questions relies on the integrity of TC, and amnesics typically either answer “I don’t know” or confabulate to these questions.
– neurobiologically grounded in a specific neural structure, the hippocampus;
– lost in hippocampal amnesia and malfunctioning in confabulation, because it receives distorted information from more than 20 damaged, predominantly orbitofrontal areas.

Some authors have used the term TC in a loose and unspecified sense, not referring to what TC is in Dalla Barba’ formulation. This caused to some misunderstanding. In a recent work, for example, Craver et al. (2014, p. 192) argued that La Corte et al. (2011) and Dalla Barba and La Corte’s (2013) concept of TC is ambiguous, because in their definition TC “comprises many cognitive faculties, including many that are preserved in people with severe deficit in episodic memory and future thought.” TC definition was probably not sufficiently clear in Dalla Barba and La Corte (2013). In the MCTT (Dalla Barba, 2002) and in other works from Dalla Barba’s group, TC is meant to refer to individuals’ phenomenological experience of remembering their past, of being present to their present world, and to predict episodes in their future. Craver et al. 2014, p. 192) say that “If TC is defined simply as the ability to remember past personal experiences and to episodically imagine future personal experiences, then KC—the well-know amnesic patient they describe in their work—lacks TC.” Dalla Barba and co-worker’s definition of TC refer exactly to this ability and not to the ability, which is preserved in amnesics, to access information concerning personal past, present and future. Craver et al. (2014) argue that episodic amnesia can spare many aspects of TC. KC had preserved semantic knowledge of time, he had little or no difficulties with physical, or chronological time and preserved order of succession judgement. In other words, KC had good knowledge of time:

“KC consciously understands the past, present and future, is aware of the fact that he has a past, present and future, and appreciates the implications of an event’s being in the past, present or future (such as the temporal asymmetry of causation and the irrevocability of the past). If KC is trapped in the present, he is trapped there with an awareness of his past, present and future, that is, with temporal consciousness” (Craver et al., 2014, p. 193).

Here and throughout their work the authors mistake KC (see above) for TC. An individual can be conscious *of* time, without having TC. KC allows individuals to know many things about personal time, to be aware that they have a past and a future, to arrange personal episodes along a timeline, to have attitudes about time, to make value judgements involving time, to know what regret is and to anticipate it. These operations are spared in KC, i.e., KC (and personal semantics, see above) is preserved. Yet none of these operations of KC grants the possibility of having the phenomenological experience of remembering their past, of being present to their present world, and of predicting episodes in their personal future, because these, according to the MCTT, are operations of TC, which is exactly what KC lacks. Knowledge of autobiographical facts, the ability to order autobiographical events on a timeline are preserved in most amnesics, but, as far as they lack phenomenological experience of remembering, they are not included in Dalla Barba’s concept of TC. Autobiographical memories can be retrieved either in the mode of TC, i.e., with the phenomenological experience of remembering a specific personal past episode, or in the mode of KC, without the phenomenological experience of remembering a specific personal past episode. All KC’s time-related spared abilities that Craver and colleagues attribute to aspects of TC are indeed aspects of KC (Dalla Barba, 2002). Patient RM, for example, was a young girl suffering from isolated retrograde amnesia, who had detailed knowledge of her autobiography, but who didn’t have any phenomenological recollective experience of the episodes she could retrieve in the mode of KC (Dalla Barba et al., 1997c). This is an example of how autobiographical episodes can be retrieved in the mode of KC.

These apparent criticisms have forced us to recognize that our characterization of the phenomenon was imprecise and encompassed a wide range of temporal competencies that, in fact, are preserved in episodic amnesia.

Now, the next question is, to what extent the ideas described so far are compatible with existing theories on memory and confabulation?

A number of hypotheses have been proposed to account for confabulation.

The gap-filling account traditionally considers confabulation a more or less intentional desire to fill gaps in memory to avoid embarrassment (Bonhoeffer, 1904; Pick, 1905; Bleuler, 1949). This hypothesis has been disconfirmed by data showing that patients do not confabulate in any domain in which their memory is faulty or when they are asked to answer questions for which they have a mandatory gap in memory and for which both non-confabulating amnesics and normal subjects the “normal” response is “I don’t Know” (“What did you do on March 13, 1985?”, or “Who was the President of Mexico in 1975?” (Dalla Barba, 1993a; Dalla Barba et al., 1997b; Dalla Barba and Decaix, 2009; Schnider et al., 1996a). A related hypothesis is the one initially proposed by Conway and Tacchi (1996) and later developed by Fotopoulou et al. (2004, 2007), which states that confabulations are often motivated, guided by a wishful thinking, in order to embellish the patient’s current situation. An argument in favor of this idea has been that confabulations often have a positive emotional content. Motivation and positive content of confabulation certainly occur in some cases and is not incompatible with the ideas we propose here. However, confabulations with negative, dark content have also been reported (Dalla Barba et al., 1998).

The executive dysfunction hypothesis has also been proposed to explain confabulation (Stuss et al., 1978; Kapur and Coughlan, 1980; Moscovitch and Melo, 1997). However, it has been shown that an executive/frontal dysfunction is neither necessary, nor sufficient for confabulation to occur (Dalla Barba et al., 1990, 1997a, 1999; Delbecq-Derouesné et al., 1990; Dalla Barba, 1993a).

Another group of theories proposes that confabulation is the result of a failure of monitoring processes. These theories hold that in confabulation processes involved in the evocation and verification of memories are impaired (Moscovitch, 1989, 1995; Johnson, 1991; Burgess and Shallice, 1996b; Moscovitch and Melo, 1997; Gilboa et al., 2006a; Schnider, 2008).

According to Johnson et al. (1997) confabulation reflects poor source monitoring, or reality monitoring, i.e., deciding whether a memory is a trace of something that actually happened to you, or is a memory of an imagined event. Impaired reality monitoring due to frontal damage would result in confabulation. However, it has been shown that reality monitoring was equally disrupted in a confabulatory patient and in non-confabulating patients with frontal lobe damage (Johnson et al., 1997). Accordingly, a reality monitoring deficit may occur with confabulation but is not the only factor involved in the genesis of confabulation (Johnson et al., 1997).

According to Moscovitch and colleagues (Moscovitch, 1989, 1995; Moscovitch and Melo, 1997; Gilboa et al., 2006a) confabulation results from the disruption of strategic retrieval, a monitoring, effortful, self-initiated cognitive process. If strategic retrieval is impaired due to orbitofrontal damage, memories are retrieved associatively, i.e., automatically, so that the first idea that comes to mind is accepted as a true memory, although it is actually a confabulation. A similar account of confabulation is proposed by other models (e.g., Burgess and Shallice, 1996a).

Within the group of monitoring theories of confabulation, Schnider and colleagues have proposed that confabulation is due to reality confusion resulting from a deficit of reality filtering following lesions to the posterior OFC (Brodman’s area 13) or structures directly connected with it (Schnider and Ptak, 1999; Gilboa and Moscovitch, 2002). According to Schnider and colleagues, reality filtering describes a memory control process necessary to maintain thinking and behavior in phase with reality (Schnider, 2008). It depends on orbitofrontal area 13 and connected subcortical structures, is electrocortically expressed at 200–300 ms after evocation of a memory and is under dopaminergic modulation. They further argue that reality filtering can be traced back to extinction capacity, i.e., the ability to learn when previously valid anticipations no longer apply to current reality and behavior needs to be adapted (Nahum et al., 2010, 2011). A problem concerning this model, is the claim that filtering (monitoring) of evoked memories occurs at 200–300 ms. In our view it is quite difficult to understand how a memory, for example “Last night I had dinner at the restaurant”, can be verified and subsequently accepted or rejected in such a short time. An additional problem is that Schnider’s and colleagues experiments on confabulation are run in a time window of minutes (up to 30 min), whereas patients confabulate for episodes that occurred well beyond Schnider’s and colleagues experimental time setting. Accordingly, it is questionable whether their experimental reduction can really be informative on the neurobiological and cognitive mechanisms underlying confabulation.

Taken together, theories that emphasize the disruption of monitoring/filtering processes in the origin of confabulation attribute to the frontal lobe, namely to the OFC, the role of searching and evaluating memories and information in the hippocampus and in the TPC. However, it is not specified on what theoretical basis the OFC would operate the search and evaluation of memory and information in the posterior part of the brain. In these theories the frontal cortex is assumed to have a subjective intentional life. Dalla Barba has indicated this as “the fallacy of the homunculus” (Dalla Barba, 2001, 2002), that is the idea that an unconscious subject, an *homunculus*, makes a selection between true and false memories and provide consciousness only with true memories. It is well known that patients with orbitofrontal lesions suffer from disinhibition, which involves not only memory, but the patient’s entire behavior. Therefore it is reasonable to think that a disrupted OFC and related structures, provide the hippocampus, through the Papez circuit, with already distorted information that are temporalized as true memories and informations. This interpretation is more economical in that it avoids the involvement of strategic retrieval and monitoring processes, which, at the state of the art, need to be more firmly theoretically grounded.

To summarize, the model described in this work is:

1. Compatible with current knowledge about functions and specialization of the human hippocampus. In particular, pattern separation and completion (e.g., Rolls, 2013; Deuker et al., 2014) and fine-grained local representations (e.g., Poppenk et al., 2013) may well be properties expressed in TC.
2. Internally coherent and theoretically grounded, without the need to postulate the existence of unconscious explanatory idols (Dalla Barba, 2009) like monitoring theories do.
3. Powerful in that it accounts in neurocognitive terms for amnesias and different forms of confabulation.

Unlike models which, being based on unconscious explanatory idols, are impermeable to scientific investigation, the account proposed here is scientifically falsifiable. Specifically, the present model will be disconfirmed if converging evidence will show that:

1. Patients with complete bilateral hippocampal damage are able to answer questions tapping TC. Questions like: “Do you remember what you had for dinner last night, the last time you went to the restaurant, the last time you went for a swim?”, or “Can you predict when you will be going to the restaurant, for a swim next time?”
2. Patients with complete bilateral hippocampal destruction confabulate.
3. Patients who are amnesic for their personal past have phenomenological experience of their future.
4. Patients who confabulate in remembering their personal past do not confabulate when having phenomenological experience of their future.

Until converging counterevidence disconfirming the model will be provided, this, together with its central assumptions, should be considered a valuable account of existing knowledge and information concerning normal and pathological memory and its neurobiological bases.

## Funding

Grant sponsor: Agence Nationale de la Recherche, Grant Number: ANR-09-EMER-006.

## Conflict of Interest Statement

The authors declare that the research was conducted in the absence of any commercial or financial relationships that could be construed as a potential conflict of interest.
